# Long-Term Quality of Life Following Transthoracic and Transhiatal Esophagectomy for Esophageal Cancer

**DOI:** 10.1007/s11605-020-04783-4

**Published:** 2020-09-09

**Authors:** E. Jezerskyte, L. M. Saadeh, E. R. C. Hagens, M. A. G. Sprangers, L. Noteboom, H. W. M. van Laarhoven, W. J. Eshuis, M. C. C. M. Hulshof, M. I. van Berge Henegouwen, S. S. Gisbertz

**Affiliations:** 1 Amsterdam UMC, location AMC, Department of Surgery, Cancer Center Amsterdam, University of Amsterdam, Amsterdam, The Netherlands; 2 General Surgery Unit, University Hospital of Padua, Padua, Italy; 3 Amsterdam UMC, location AMC, Department of Medical Psychology, University of Amsterdam, Amsterdam, The Netherlands; 4 Amsterdam UMC, location AMC, Department of Medical Oncology, Cancer Center Amsterdam, University of Amsterdam, Amsterdam, The Netherlands; 5 Amsterdam UMC, location AMC, Department of Radiotherapy, University of Amsterdam, Amsterdam, The Netherlands

**Keywords:** Esophagectomy, Quality of life, Esophageal neoplasms

## Abstract

**Background:**

The impact of transthoracic (TTE) and transhiatal esophagectomy (THE) on long-term health-related quality of life (HR-QoL) in patients with distal esophageal or gastro-esophageal junction (GEJ) cancer has been studied with variable results. This study investigates long-term HR-QoL in patients having undergone TTE or THE.

**Methods:**

Disease-free patients after TTE or THE for distal esophageal or GEJ cancer with a follow-up > 2 years were included. Patients who visited the outpatient clinic of a tertiary referral center between 2014 and 2018 were asked to complete EORTC-QLQ-C30 and EORTC-QLQ-OG25 questionnaires. Uni- and multivariable linear regression analysis of HR-QoL was performed in all patients and in subgroups of minimally invasive esophagectomy and neoadjuvant therapy.

**Results:**

A total of 132 patients after TTE and 56 after THE were included. When compared with the general population, all patients reported worse HR-QoL in ‘role functioning’ and ‘social functioning’ and in a range of disease- and/or treatment-specific symptoms. The only significant difference between TTE and THE was a better HR-QoL score for “hair loss” following TTE (ß = 29.4,95%CI = -49.108 – −9.671, *p* = 0.016). Subgroup analysis of minimally invasively operated patients showed better scores in “physical functioning” following TTE (ß = 13.8,95%CI = 2.755–24.933, *p* = 0.030). No significant differences in HR-QoL were found between TTE and THE after neoadjuvant therapy**.**

**Conclusion:**

Long-term HR-QoL is largely comparable in disease-free patients following TTE or THE for distal esophageal or GEJ cancer. If there were differences between the surgical groups, they were in favor of TTE. These findings may aid in preoperative counseling of patients with esophageal or GEJ cancer.

**Electronic supplementary material:**

The online version of this article (10.1007/s11605-020-04783-4) contains supplementary material, which is available to authorized users.

## Introduction

Treatment of esophageal cancer usually consists of surgery in combination with (neo)adjuvant chemo(radio)therapy. Both a transhiatal (THE) and a transthoracic (TTE) esophagectomy may be feasible in distal esophageal and gastro-esophageal (GEJ) junction cancer. As survival of patients with esophageal cancer improves, the long-term health-related quality of life (HR-QoL) is becoming increasingly more important. In a randomized controlled trial (RCT), no significant survival differences were found between TTE and THE, although more lymph nodes were resected and more pulmonary complications were documented in the TTE group.^1^,^2^

A number of studies have assessed HR-QoL following TTE and THE and show diverse results.^2^–^6^ Some studies, including the previously mentioned RCT, did not find differences in HR-QoL,^3^,^5^,^6^ whereas one study showed worse long-term HR-QoL following TTE in comparison with THE.^4^ The results of these studies do not completely apply to current practice, as most were performed before the implementation of minimally invasive surgery and neoadjuvant therapy. For example, minimally invasive TTE results in fewer pulmonary complications and is associated with less postoperative pain, which in turn was found to positively affect HR-QoL.^7^,^8^ Furthermore, HR-QoL was found to decline during neoadjuvant chemoradiotherapy in patients with esophageal cancer,^9^ but no negative impact of neoadjuvant therapy has been found on postoperative HR-QoL after a follow-up of 12 months.^10^–^14^ However, these findings are based on RCT’s with pre-selected patients. The rationale for this study is to investigate long-term HR-QoL in esophageal cancer patients following esophagectomy from a naturally occurring sample in the era where minimally invasive surgery and neoadjuvant therapy have become standard treatment.

The aim of this study is to investigate long-term HR-QoL in disease-free patients after TTE and THE for distal esophageal and GEJ cancer in a tertiary referral center. Secondary aim is to compare long-term HR-QoL between TTE and THE in the “minimally invasive” and “neoadjuvant therapy” subgroups.

## Methods

### Study Design, Patients, and Follow-Up

In this prospective cohort study, patients were enrolled between October 2014 and October 2018 in Amsterdam UMC, location Academic Medical Center (AMC). Patients were asked to participate if they had undergone a THE or a TTE for a distal esophageal or GEJ cancer between 2006 and 2016. Included patients completed the European Organization for Research and Treatment of Cancer (EORTC) quality-of-life questionnaires during outpatient clinic visits. Essentially, all included patients with a distal esophageal or GEJ cancer could technically have undergone both a TTE or a THE. Patients with a recurrence during follow-up, having undergone salvage esophagectomy and/or jejunal or colonic interposition, and patients who died were excluded from this study. Also, patients with mediastinal lymph node metastases above the level of the pulmonary vein were excluded, as in these patients only a TTE can be performed. Clinical information and reason for rejection of patients who declined participation were not registered, due to data protection regulations.^15^–^17^

Patients were seen at regular intervals at the outpatient clinic until 5 years after surgery or longer if indicated. Imaging was only performed if a recurrence was clinically suspected, which is in accordance with the Dutch guideline.^18^

The Institutional Review Board of Amsterdam UMC waived ethical approval. Patients gave oral informed consent. This article ensured accurate reporting by adhering to the STROBE checklist.^19^

### Neoadjuvant Chemoradiotherapy or Perioperative Chemotherapy and Surgery

In the AMC, individual patient treatment is decided upon during a weekly multi-disciplinary team meeting at the Gastrointestinal Oncology Center Amsterdam (GIOCA). Neoadjuvant therapy regimens for ≥cT2N0-3 M0 or cT1N+ cancers in the period under study consisted of neoadjuvant chemoradiotherapy according to the CROSS scheme or, if there is more than 2-cm tumor involvement of the stomach, perioperative chemotherapy according to the MAGIC scheme.^20^ A THE with a gastric tube reconstruction used to be the preferred approach for distal esophageal and GEJ cancer but was gradually replaced by the transthoracic approach during the study period, because of the more radical lymphadenectomy by TTE. In the studied period, both procedures were still carried out regularly. THE and TTE were either performed open or minimally invasively, depending on patient and tumor characteristics and time period (minimally invasive surgery was introduced in 2010). In THE, a 1-field lymphadenectomy was performed with extension of the field to the lower mediastinum (lymph node stations according to the 8th edition of the AJCC: 8Lo, 9, 15–20). During TTE, a 2-field lymphadenectomy was performed, including the paratracheal lymph node stations (lymph node stations 4, 5, 7, 8 M, 8Lo, 9, 15–20, and 2 and 8Up on indication). During TTE either a cervical or intrathoracic anastomosis was performed, depending on either tumor characteristics or time period (the intrathoracic anastomosis was introduced in 2013).

### Background, Clinical, and Postoperative Morbidity Variables

Clinical data were retrieved from a prospectively maintained upper gastrointestinal surgery database at the Amsterdam UMC, location AMC. The included background and clinical variables were age, gender, follow-up (months), tumor location (distal esophagus or GEJ), comorbidities (cardiovascular, pulmonary or metabolic), ASA classification, (neo)adjuvant therapy (chemotherapy or chemoradiotherapy), surgical approach (open or minimally invasive), cTNM stage, histologic tumor type (adenocarcinoma, squamous cell carcinoma or other), (y)pTNM stage, R0 resection rate, number of retrieved (positive) lymph nodes, and tumor response after neoadjuvant therapy according to tumor regression grading (TRG).^21^ Postoperative morbidity variables included Clavien-Dindo classification (grade 5 was excluded from this study), and the following complications: atrial fibrillation, anastomotic leakage, pneumonia (these are the complications with the highest prevalence)^22^ and other complications (wound infection, intra-abdominal abscess, sepsis, recurrent nerve injury, intrathoracic hernia, empyema, pulmonary embolus, pneumothorax) according to the Esophagectomy Complications Consensus Group (ECCG).^23^

### Health-Related Quality of Life

The cancer-specific (EORTC QLQ-C30) and gastro-esophageal site-specific (EORTC QLQ-OG25) questionnaires were used, which are validated for cancer patients and gastro-esophageal cancer patients, respectively.^24^–^26^ The EORTC QLQ-C30 contains 30 questions, out of which 15 multi- and single-scale domains are generated: one “global health” domain, five functional domains (“physical,” “role,” “social,” “cognitive,” and “emotional functioning”), and nine symptom domains (“fatigue,” “nausea and vomiting,” “pain,” “dyspnea,” “insomnia,” “appetite loss,” “constipation,” “diarrhea,” and “financial difficulties”). The EORTC QLQ-OG25 contains 25 questions of which 16 multi- and single-scale domains are generated: one functional domain (“body image”) and 15 symptom domains (”dysphagia,” “reflux,” “odynophagia,” and “problems with eating with others,” “pain and discomfort,” “anxiety,” “problems with eating,” “dry mouth,” “trouble with taste,” “trouble swallowing saliva,” “choking when swallowing,” “trouble with coughing,” “trouble talking,” “weight loss,” and “hair loss”).

Both questionnaires use a Likert scale of four points with answers ranging from 1 “not at all” to 4 “very much,” except for the two questions about global HR-QoL, which employ a response scale ranging from 1 “very poor” to 7 “excellent.” Following the scoring manual of EORTC QoL Group, all answers were linearly transformed into domain scores ranging from 0 to 100.^27^ A high score in “global health” and functional domains represents better HR-QoL and functioning, in contrast to symptom domains where a low score represents a low level of symptomatology and hence better HR-QoL.

### Statistical Analysis

First, the mean HR-QoL domain scores of the total study group (TTE and THE combined) with those of the general population were compared, based on the EORTC reference values manual.^28^,^29^ A mean score difference of more than 10 points was considered meaningful. Categorical variables (i.e., postoperative morbidity, patient and tumor characteristics) were subsequently analyzed using Chi^2^ or Fisher’s exact tests. In case of continuous variables, Student’s *t* test (for normally distributed variables) or Mann-Whitney U test (for not normally distributed variables) were used.

For the analysis of the difference in HR-QoL domain scores between TTE and THE, univariable and multivariable linear regression analysis was used. HR-QoL domain scores were entered in the multivariable analysis if a *p* value of <0.10 was reached in univariable analysis. In multivariable analysis, HR-QoL domain scores were standardly corrected for the possible confounders age and gender. Also, all background variables with a *p* value difference of < 0.10 between TTE and THE groups were considered candidate confounders. A variable was added to the multivariable analysis as a confounder if it caused clinically relevant effect (a change of > 10% in regression coefficient). Furthermore, two additional subgroup univariable and multivariable linear regression analyses of HR-QoL domain scores were performed for patients operated minimally invasively (TTE versus THE) and for patients treated with neoadjuvant therapy (TTE versus THE). In addition, to investigate whether the level of the anastomosis in the TTE group influenced results, an additional subgroup analysis of HR-QoL domain scores was performed for patients in the TTE group with either a cervical or an intrathoracic anastomosis. Two-sided testing was performed. A *p* value of < 0.05 was considered as statistically significant. A Bonferroni correction for multiple testing was performed for all HR-QoL domains that were entered in the multivariable analysis by multiplying the *p* value by the number of tests performed in the multivariable analysis. Furthermore, mean difference (ß) in HR-QoL domain scores between two groups of 10 points or more was considered clinically relevant according to the EORTC guideline.^30^ Statistical analyses were performed in SPSS Statistics version 24.

## Results

### Demographics and Cohort Features

**Fig. 1:**
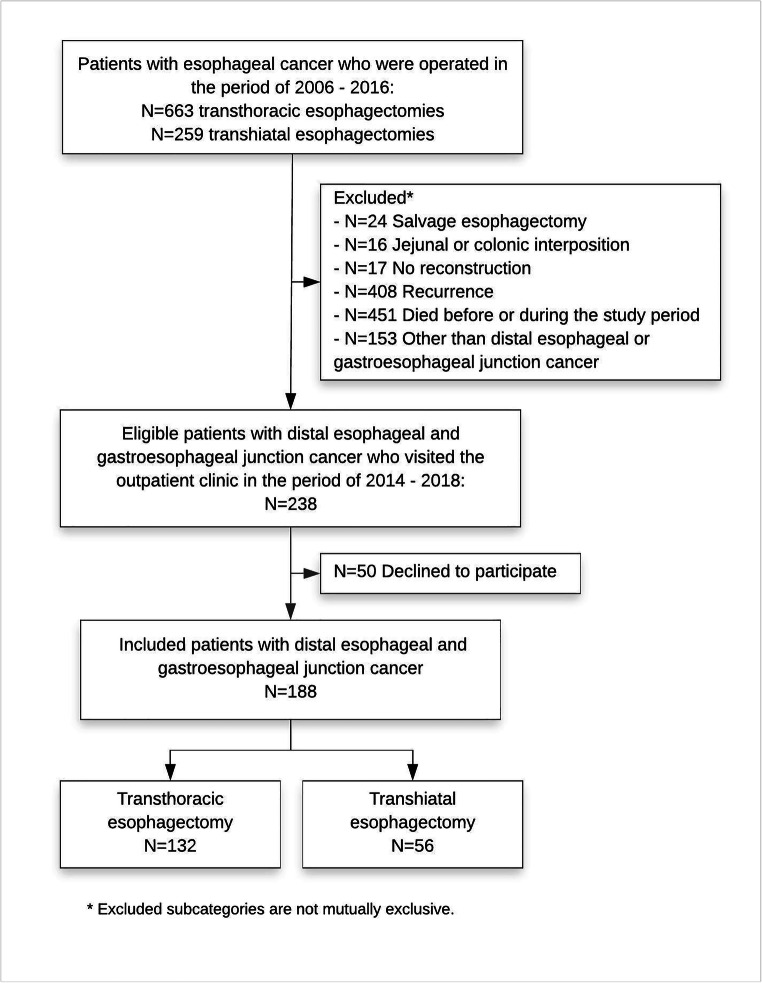
Study flow chart

There were 238 eligible patients who visited the outpatient clinic during the inclusion period. Of these 238 patients, 188 completed the questionnaires (response rate 78.9%): 132 patients after TTE and 56 after THE (Fig. 1). Median follow-up was significantly different between the two groups: 3.2 years [IQR 2.3–4.3] in the TTE group and 4.7 years [IQR 3.4–6.2] in the THE group (*p* < 0.001). Median age was significantly higher in THE group (66 years [IQR 61–72] compared with TTE group (64 years [IQR 58–68], *p* = 0.024). The majority of patients had ASA classification of 2, and there was no significant difference in comorbidities (cardiovascular, pulmonary, or metabolic). Neoadjuvant chemoradiotherapy was administered more often in the TTE group (87.9% versus 50%; *p* = 0.001). Also, TTE was performed significantly more often minimally invasively compared with THE (84.8% versus 39.3%; *p* < 0.001). In the TTE group, a cervical anastomosis was performed in 59 (44.7%) patients; in the other 73 (55.3%), an intrathoracic anastomosis was performed. The majority of patients had an adenocarcinoma (87.8%). A significantly higher number of resected lymph nodes was found after TTE (median 26 [IQR 20–34]) compared with THE (median 18 [IQR 14–24], *p* < 0.001), but the number of positive lymph nodes and tumor-free resection margins were not significantly different between groups (Table 1).

**Table 1:** Baseline characteristics of patients with distal esophageal or junctional cancer operated with transthoracic or transhiatal esophagectomy between 2006 and 2016

			TTE		THE		*p* value
N			132		56		
Age (median (IQR), y)			64	(58–68)	66	(61–72)	**0.024**
Gender	Male		110	(83.3)	41	(73.2)	0.111
Tumor location	Distal esophagus		121	(91.7)	31	(55.4)	0.088
	Gastro-esophageal junction		11	(8.3)	25	(44.6)	
Comorbidity	No		70	(53.0)	27	(48.2)	0.546
	Cardiovascular		52	(39.4)	22	(39.3)	0.989
	Pulmonary		9	(6.8)	6	(10.7)	0.385
	Metabolic		14	(10.6)	4	(7.1)	0.460
ASA classification	1		41	(31.1)	11	(19.6)	0.269
	2		67	(50.8)	34	(60.7)	
	3		24	(18.2)	11	(19.6)	
Neo-adjuvant therapy	No		16	(12.1)	22	(39.3)	**< 0.001**
	Yes	Chemotherapy	1	(0.8)	6	(10.7)	**0.001**
		Chemoradiotherapy	115	(87.9)	28	(50.0)	
Approach	Open		20	(15.2)	34	(60.7)	**< 0.001**
	Minimally invasive		112	(84.8)	22	(39.3)	
Location of the anastomosis	Cervical		59	(44.7)	56	(100)	N/A
	Intrathoracic		73	(55.3)	N/A	N/A	
cT	T0		4	(3.0)	1	(1.8)	0.139
	T1		12	(9.1)	11	(19.6)	
	T2		35	(26.5)	8	(14.3)	
	T3		79	(59.8)	35	(62.5)	
	T4		2	(1.5)	1	(1.8)	
cN	N0		50	(37.9)	29	(51.8)	0.191
	N1		63	(47.7)	24	(42.9)	
	N2		16	(12.1)	3	(5.4)	
	N3		3	(2.3)	0	–	
Adjuvant therapy	No		115	(87.1)	47	(83.9)	0.562
	Yes	Chemotherapy	13	(86.7)	8	(88.9)	1.000
		Chemoradiotherapy	4	(13.3)	1	(1.8)	
Histologic type	Adenocarcinoma		114	(86.4)	51	(91.1)	0.737
	Squamous cell carcinoma		13	(9.8)	4	(7.1)	
	Other		5	(3.8)	1	(1.8)	
(y)pT	T0		41	(31.1)	9	(16.1)	**0.026**
	T1		33	(25.0)	10	(17.9)	
	T2		18	(13.6)	8	(14.3)	
	T3		40	(30.3)	29	(51.8)	
(y)pN	N0		95	(72.0)	37	(66.1)	0.486
	N1		27	(20.5)	17	(30.4)	
	N2		6	(4.5)	1	(1.8)	
	N3		4	(3.0)	1	(1.8)	
pM	M1		1	(0.8)	0	–	1.000
Radicality	R0		132	(100)	56	(100)	N/A
Lymph nodes (median (IQR))			26	(20–34)	18	(14–24)	**< 0.001**
Lymph node metastases (median (IQR))			0	(0–1)	0	(0–1)	0.179
Tumor response after neoadjuvant therapy according to TRG	No response		5	(4.3)	2	(6.5)	0.215
	Intermediate response		71	(61.7)	25	(80.6)	
	Complete response		39	(33.9)	4	(12.9)	

Data are presented as n (%) unless otherwise indicated. *IQR* interquartile range, *y* year. American Society of Anesthesiologists (ASA) classification. c/pTNM tumor staging classification. N/A = not applicable. TRG = tumor response grading. Bold values represent significance

Anastomotic leakage, pneumonia, atrial fibrillation and other postoperative complications did not occur significantly differently between the two groups. Furthermore, no significant difference was found in Clavien-Dindo grade between TTE and THE (Table 2).

**Table 2:** Postoperative morbidity of patients with a distal esophageal or gastro-esophageal junction cancer operated with either transthoracic or transhiatal esophagectomy

			TTE (*N* = 132)		THE (*N* = 56)		*p* value
Postoperative complications	No		71	(53.8)	26	(46.4)	0.356
	Yes		61	(46.2)	30	(53.6)	
		Anastomotic leakage	18	(13.7)	13	(23.2)	0.111
		Pneumonia	19	(14.4)	7	(12.5)	0.731
		Atrial fibrillation	27	(20.6)	6	(10.9)	0.114
		Other	51	(38.6)	20	(35.7)	0.705
Clavien-Dindo classification	Grade 0		65	(49.2)	26	(46.4)	0.178
	Grade 1		7	(5.3)	6	(10.7)	
	Grade 2		25	(18.9)	15	(26.8)	
	Grade 3A		14	(10.6)	4	(7.1)	
	Grade 3B		0	0	2	(3.6)	
	Grade 4A		18	(13.6)	3	(5.4)	
	Grade 4B		3	(2.3)	0	0	

Data are presented as n (%) unless otherwise indicated

### Long-Term HR-QoL Following TTE or THE

All patients reported worse HR-QoL compared with the general population in “role functioning” (mean difference 10.4), “social functioning” (mean difference 11.6), “nausea and vomiting” (mean difference 10.3), “dyspnea” (mean difference 16.6), “appetite loss” (mean difference 11.5), “financial difficulties” (mean difference 20.5), “dysphagia” (mean difference 11.4), “eating difficulties” (mean difference 23.0), “eating with others difficulties” (mean difference 13.5), “reflux” (mean difference 15.8), “choking when swallowing” (mean difference 13.6), “trouble with coughing” (mean difference 16.4) and “weight loss” (mean difference 19.9).^28^,^29^

After univariable linear regression analysis of all HR-QoL domains between TTE and THE, a *p* value of <0.10 was found in “emotional functioning,” “social functioning,” “constipation,” and “hair loss.” Background variables age, gender, tumor location, neoadjuvant treatment, type of neoadjuvant therapy, surgical approach, **(y)**pT stage, lymph node yield, and follow-up were selected as confounders for multivariable analysis. After multivariable analysis and Bonferroni correction, significantly fewer “problems with hair loss” (mean score difference = 29.4, 95%CI = -49.108 – −9.671, *p* = 0.016) were found after TTE compared with THE. This difference in mean scores was clinically relevant with 29.4 points difference. Also, a clinically relevant difference in mean scores of 15.0 points was found in “social functioning” domain. However, this difference was not statistically significant (Table 3 and supplementary Table 1).

**Table 3:** Univariable and multivariable linear regression analysis of HR-QoL comparing transthoracic and transhiatal esophagectomy

			Univariable analysis				Multivariable analysis†				
	Transthoracic esophagectomy Mean (SD)	Transhiatal esophagectomy Mean (SD)	B	95%CI		*p* value	B	95%CI		*p* value	Corrected *p* value‡
	*n* = 132	*n* = 56		Lower	Upper			Lower	Upper		
EORTC QLQ-C30											
Global Health	72.7 (19.1)	76.71 (23.8)	4.0	−3.259	11.230	0.277					
Functioning											
Physical functioning	81.7 (19.6)	77.9 (21.2)	−3.9	−10.255	2.377	0.220					
Role functioning	74.3 (27.1)	74.4 (31.8)	0.1	−9.009	9.164	0.987					
Emotional functioning	78.0 (23.6)	85.9 (20.8)	8.0	0.702	15.226	0.032*	−3.0	−13.683	7.761	0.586	2.344
Cognitive functioning	82.9 (23.1)	86.4 (13.8)	3.5	−1.896	8.952	0.201					
Social functioning	78.8 (25.8)	69.0 (35.5)	−10.0	−20.333	0.570	0.064*	15.0	2.724	27.230	**0.017**	0.068
Symptom scores											
Fatigue	32.6 (27.0)	30.2 (29.1)	−2.4	−11.138	6.262	0.581					
Nausea and vomiting	14.6 (22.8)	12.7 (19.6)	−2.0	−8.871	4.906	0.571					
Pain	18.1 (24.7)	14.3 (23.9)	−3.8	−11.475	3.914	0.334					
Dyspnea	29.8 (28.9)	25 (30.7)	−4.8	−14.058	4.462	0.308					
Insomnia	26.3 (31.3)	20.8 (26.6)	−5.5	−14.937	3.952	0.253					
Appetite loss	19.9 (30.6)	13.9 (27.7)	−6.0	−15.423	3.480	0.214					
Constipation	7.7 (15.8)	14.8 (25.6)	7.1	−0.363	14.608	0.062*	−3.1	−11.794	5.502	0.473	1.892
Diarrhea	15.4 (21.2)	18.5 (26.4)	3.1	−4.154	10.383	0.399					
Financial difficulties	29.8 (29.7)	30.3 (40.7)	0.5	−11.595	12.585	0.935					
EORTC QLQ-OG25											
Functioning											
Body image	77.3 (33.2)	69.3 (37.7)	−8.0	−18.990	2.924	0.150					
Symptom scores											
Dysphagia	10.9 (16.5)	15.3 (18.4)	4.4	−1.010	9.774	0.111					
Eating	26.3 (24.7)	25.1 (28.6)	−1.2	−9.389	6.982	0.772					
Reflux	22.1 (27.9)	23.4 (28.1)	1.3	−7.604	10.104	0.781					
Odynophagia	10.0 (20.4)	10.4 (16.7)	0.4	−5.728	6.534	0.897					
Pain and discomfort	15.9 (26.6)	17.3 (22.2)	1.4	−6.650	9.415	0.735					
Anxiety	31.4 (29.1)	26.3 (31.6)	−5.1	−14.525	4.345	0.289					
Eating with others	14.2 (25.0)	16.4 (27.9)	2.2	−6.063	10.444	0.601					
Dry mouth	18.7 (26.6)	18.5 (25.4)	−0.2	−8.530	8.101	0.960					
Trouble with taste	13.0 (23.1)	8.9 (19.6)	−4.0	−11.029	2.961	0.257					
Trouble swallowing saliva	10.5 (22.2)	10.7 (20.2)	0.2	−6.567	7.065	0.943					
Choked when swallowing	15.8 (24.4)	20.8 (28.1)	5.1	−3.018	13.189	0.217					
Trouble with coughing	31.8 (31.7)	26.2 (32.2)	−5.6	−15.646	4.510	0.277					
Trouble talking	8.5 (18.5)	7.1 (16.5)	−1.3	−6.980	4.336	0.645					
Weight loss	23.7 (31.1)	16.3 (29.4)	−7.4	−17.705	2.954	0.161					
Hair loss	15.9 (28.9)	31.3 (34.9)	15.4	−0.636	31.431	0.060*	−29.4	−49.108	−9.671	**0.004**	**0.016**

Regression coefficient (B) with 95% confidence interval (CI) are shown for univariable and multivariable analysis. † = Corrected for confounders (Supplementary Table 1). * = Health related quality of life (HR-QoL) domains with *p* < 0.1 in univariable analysis were entered in multivariable analysis

Bold *p* values (*p* <  0.05) represent significance

‡ = *p* value corrected for multiple testing according to Bonferroni method

### Long-Term HR-QoL Following Minimally Invasive Surgery

A total of 134 patients were operated minimally invasively: 112 received a minimally invasive TTE and 22 received a minimally invasive THE. After univariable analysis of all HR-QoL domains, a *p* value of < 0.10 was found in “physical functioning” and “trouble talking” domains. Background variables age, gender, neoadjuvant treatment (yes/no), type of neoadjuvant therapy, cN stage, and lymph node yield were selected as confounders (*p* value < 0.10 in univariable analyses) for multivariable analysis. After multivariable analysis and Bonferroni correction, only “physical functioning” was found to be significantly better after minimally invasive TTE compared with minimally invasive THE (mean score difference = 13.8, 95%CI = 2.755–24.933, *p* = 0.030) with a clinically relevant difference in mean scores of 13.8 points (Supplementary Tables 2 and 3).

### Long-Term HR-QoL Following Neoadjuvant Therapy

A total of 116 patients in TTE group and 34 patients in THE group received neoadjuvant treatment. Background variables age, gender, ASA classification, surgical approach, pT stage, lymph node yield, and follow-up were selected as confounders for the multivariable linear regression analysis. “Social functioning,” “insomnia,” “constipation,” and “choked when swallowing” domains were entered in the multivariable linear regression analysis as these domains had a *p* value < 0.10 in univariable analysis. A clinically relevant difference in mean scores of 13.4 points was found in “social functioning” domain between patients after neoadjuvant therapy and TTE compared with patients after neoadjuvant therapy and THE. However, this difference was not statistically significant (Supplementary Tables 2 and 4).

### Long-Term HR-QoL Following a Cervical or Intrathoracic Anastomosis after TTE

A total of 59 patients with a cervical anastomosis and 73 patients with an intrathoracic anastomosis were included in the TTE group. Background variables age, gender, follow-up, diabetes, tumor location, surgical approach, cN stage, histology, (positive) lymph node yield, and adjuvant therapy were selected as confounders for the multivariable linear regression analysis. After univariable analysis only in “fatigue” score, a *p* < 0.1 was found. After multivariable analysis, no significant or clinically relevant differences were found in patients with either a cervical or intrathoracic anastomosis after TTE (data not shown).

## Discussion

This study investigated long-term HR-QoL in disease-free patients following either a TTE or a THE for distal esophageal and GEJ cancer. All patients reported impaired quality of life compared with the general population in “role functioning” and “social functioning,” and as expected, in a range of disease- and/or treatment-specific symptoms. The long-term HR-QoL was, in general, not significantly different between patients who had undergone TTE or THE. Patients following TTE reported fewer problems with hair loss compared with THE. Subgroup analysis of minimally invasively operated patients showed better physical functioning in patients following TTE than THE. Subgroup analysis of patients following neoadjuvant therapy showed no differences in HR-QoL between TTE and THE. These few differences in HR-QoL do not have a decisive effect when choosing between the two surgical approaches. However, hair loss and physical functioning can impact daily social and physical activities adversely and may have a major impact on patients’ well-being. Therefore, patients should be informed of these possible long-term effects on HR-QoL before surgery.

Earlier studies reported that the inevitable postoperative decrease in HR-QoL is restored within 1 year after esophagectomy in disease-free patients.^31^,^32^ This is also seen in patients following TTE and THE; as overall, no significant differences in HR-QoL have been reported that last up to 1^6^ or 3 years postoperatively.^3^,^5^ Only one study reported more “nausea and vomiting,” “dyspnea,” and “constipation” 12 months after open TTE compared with open THE 4. However, the results of these studies may not be completely applicable to current clinical practice as they were performed before the implementation of neoadjuvant therapy and minimally invasive esophagectomy in the treatment of esophageal cancer.^7^,^20^ In our study, patients reported less problems with hair loss after TTE compared with THE. This difference could be due to the administration of less chemotherapy in the TTE group. Similarly, a recent meta-analysis showed that patients reported more hair loss after chemotherapy and esophagectomy compared with patients after chemoradiotherapy and esophagectomy.^33^

When minimally invasive esophagectomy is compared with open esophagectomy, better HR-QoL is found in “global QoL,” “physical functioning,” “fatigue,” and “pain” domains at 3 months following a minimally invasive esophagectomy.^34^ However, no difference in HR-QoL was observed after a follow-up of 12 months. In our study no subgroup analysis of HR-QoL could be performed between minimally invasive esophagectomy and open esophagectomy due to the small number of patients following open esophagectomy (*N* = 20 in TTE group and *N* = 34 in THE group). However, a subgroup analysis was performed for all minimally invasively operated patients and only one HR-QoL domain—“physical functioning”—was found to be better following TTE compared with THE. Patients in the minimally invasive TTE group were significantly younger than patients in the minimally invasive THE group (median 64 years [IQR 57–69] versus median 68 years [IQR 62–74], *p* = 0.043). We therefore corrected for age during multivariable analysis. As only a small number of patients were included in this subgroup, further investigation of HR-QoL is required employing larger sample sizes.

During neoadjuvant therapy, patients in previous studies have reported worse HR-QoL, which restores to baseline levels after completion of neoadjuvant therapy.^9^,^12^,^14^,^35^ Postoperative HR-QoL does not seem to be influenced by neoadjuvant treatment in patients with esophageal cancer.^10^–^14^ In both chemoradiotherapy and esophagectomy compared with esophagectomy alone groups, a decline in HR-QoL is seen at 3 months postoperatively.^10^,^13^ Only one study reported less dysphagia, nausea, and vomiting problems at 3 months follow-up in patients who received neoadjuvant treatment compared with surgery alone group.^12^ Overall, a gradual improvement of HR-QoL to baseline level is seen at 12 months follow-up,^10^,^35^ which remains stable the subsequent 6 years.^11^ Our results are comparable with previous studies, as no difference in HR-QoL after a follow-up of 2 years was found in subgroup analysis of patients following neoadjuvant therapy between TTE and THE.

This study has some limitations. The study is prone to selection bias because of the nature of the inclusion process. Only patients who were still actively followed up at the outpatient clinic were eligible for inclusion. Patients who died, had recurrent disease, were lost to follow-up, or who were unwilling to participate did not participate in this study, which may have led to a general bias towards the inclusion of patients who fare reasonably well. Furthermore, the results can be affected by the differences in baseline patient, treatment. and tumor characteristics between TTE and THE groups. Patients after TTE were younger, received more often neoadjuvant chemoradiotherapy, and were more often operated minimally invasively compared to patients after THE. This is mainly attributable to the time period in which patients were operated, where TTE gradually has replaced THE. This also explains the difference in follow-up. Apart from being operated upon in different time periods, the procedure of choice may have been dependent on localization and stage of disease. This may have led to additional selection bias. Also, in the TTE group, both cervical and intrathoracic anastomoses were included, what could contribute to some heterogeneity, although a recent study showed largely comparable results in HR-QoL following a transthoracic esophagectomy with either a cervical or intrathoracic anastomosis.^36^ Also in this study, subgroup analysis in patients with either a cervical or intrathoracic anastomosis following TTE did not show any significant or clinically relevant results. Furthermore, (y)pT stage and lymph node count were different between the two groups. We tried to minimize the effect of selection bias by correcting for these confounders in multivariable linear regression analysis. In addition, since this study did not employ a baseline HR-QoL measurement, we cannot exclude the possibility that QoL differed a priori between the two groups. An ongoing prospective observational cohort study of esophageal and gastric cancer patients collecting clinical data and HR-QoL prior to and following TTE and THE will shed light on possible a priori HR-QoL differences (POCOP trial, NCT 02070146).^37^ Furthermore, no formal sample size calculation was performed, and the number of statistical tests is high in relation to the sample size. We therefore used a Bonferroni correction for multiple testing. Moreover, the EORTC defined a mean difference of at least 10 points as clinically relevant. Nonetheless, we believe that the results of this study are valuable, since they provide a good insight in the well-being of disease-free patients after TTE and THE. Furthermore, this study employs a naturally occurring sample and has a relatively large sample size, a high response rate, and a long follow-up. Also, this study was the first to investigate long-term HR-QoL of patients who were operated minimally invasively and patients who received neoadjuvant therapy separately.

## Conclusion

Long-term HR-QoL results are in general not different between disease-free patients following either TTE or THE for distal esophageal or GEJ cancer. The small differences that were found were in the advantage of a TTE. These findings may aid in providing information to esophageal or GEJ cancer patients on what to expect regarding postoperative QoL. Future studies should include baseline measurements of HR-QoL. Because of the small differences in HR-QoL between THE and TTE, the oncological preference should be leading in the choice of procedure.

## Electronic supplementary material

ESM 1 (DOCX 66 kb)

## References

[1] Hulscher JB (2001). *Transthoracic versus transhiatal resection for carcinoma of the esophagus: a meta-analysis*. Ann Thorac Surg.

[2] Omloo JM (2007). *Extended transthoracic resection compared with limited transhiatal resection for adenocarcinoma of the mid/distal esophagus: five-year survival of a randomized clinical trial*. Ann Surg.

[3] de Boer AG (2004). *Quality of life after transhiatal compared with extended transthoracic resection for adenocarcinoma of the esophagus*. J Clin Oncol.

[4] Kauppila JH (2018). *Health-related quality of life after open transhiatal and transthoracic oesophagectomy for cancer*. Br J Surg.

[5] van der Schaaf M, Rutegard M, Lagergren P (2013). *The influence of surgical factors on persisting symptoms 3 years after esophageal cancer surgery: a population-based study in Sweden*. Ann Surg Oncol.

[6] Sundaram A (2012). *Survival and quality of life after minimally invasive esophagectomy: a single-surgeon experience*. Surg Endosc.

[7] Straatman J (2017). *Minimally invasive versus open esophageal resection: three-year follow-up of the previously reported randomized controlled trial: the TIME trial*. Ann Surg.

[8] Maas KW (2015). *Quality of life and late complications after minimally invasive compared to open esophagectomy: results of a randomized trial*. World J Surg.

[9] Noordman BJ (2019). *Quality of life during and after completion of neoadjuvant chemoradiotherapy for esophageal and junctional cancer*. Ann Surg Oncol.

[10] Noordman BJ (2018). *Effect of neoadjuvant chemoradiotherapy on health-related quality of life in esophageal or junctional cancer: results from the randomized CROSS trial*. J Clin Oncol.

[11] Noordman BJ (2018). *Impact of neoadjuvant chemoradiotherapy on health-related quality of life in long-term survivors of esophageal or junctional cancer: results from the randomized CROSS trial*. Ann Oncol.

[12] Blazeby JM (2005). *Health-related quality of life during neoadjuvant treatment and surgery for localized esophageal carcinoma*. Cancer.

[13] Hauser C (2015). *Does neoadjuvant treatment before oncologic esophagectomy affect the postoperative quality of life? A prospective, longitudinal outcome study*. Dis Esophagus.

[14] Reynolds JV (2006). *Prospective evaluation of quality of life in patients with localized oesophageal cancer treated by multimodality therapy or surgery alone*. Br J Surg.

[15] General Data Protection Regulation (GDPR). 2020; **Available from: **https://gdpr-info.eu/. Accessed 28 June 2020.

[16] *AMC–VUmc Research Code, version effective since 2013*. 2020; **Available from:** www.amc.nl/researchcode/. Accessed 28 June 2020.

[17] Good Clinical Practice Guidelines. 2020; **Available from:** www.ema.europa.eu. Accessed 28 June 2020.

[18] *Oncoline*. 2019; **Available from:** https://www.oncoline.nl/oesofaguscarcinoom. Accessed 28 June 2020.

[19] von Elm E (2008). *The Strengthening the Reporting of Observational Studies in Epidemiology (STROBE) statement: guidelines for reporting observational studies*. J Clin Epidemiol.

[20] Shapiro J (2015). *Neoadjuvant chemoradiotherapy plus surgery versus surgery alone for oesophageal or junctional cancer (CROSS): long-term results of a randomised controlled trial*. Lancet Oncol.

[21] Mandard AM (1994). *Pathologic assessment of tumor regression after preoperative chemoradiotherapy of esophageal carcinoma. Clinicopathologic correlations*. Cancer.

[22] van der Werf, L.R., et al., *Reporting National Outcomes After Esophagectomy and Gastrectomy According to the Esophageal Complications Consensus Group (ECCG).* Ann Surg, 2019. 10.1097/SLA.0000000000003210 30676381

[23] Low DE (2015). *International Consensus on Standardization of Data Collection for Complications Associated With Esophagectomy: Esophagectomy Complications Consensus Group (ECCG)*. Ann Surg.

[24] Lagergren P (2007). *Clinical and psychometric validation of a questionnaire module, the EORTC QLQ-OG25, to assess health-related quality of life in patients with cancer of the oesophagus, the oesophago-gastric junction and the stomach*. Eur J Cancer.

[25] Aaronson NK (1993). *The European Organization for Research and Treatment of Cancer QLQ-C30: a quality-of-life instrument for use in international clinical trials in oncology*. J Natl Cancer Inst.

[26] *EORTC*. 2019; **Available from:** http://www.eortc.org/. Accessed 28 June 2020.

[27] Fayers P, Aaronson NK, Bjordal K, Groenvold M, Curran D, Bottomley A (2001). EORTC QLQ-C30 Scoring Manual.

[28] van der Schaaf M, Derogar M, Lagergren P (2012). *Reference values of oesophago-gastric symptoms (EORTC QLQ-OG25) in a population-based setting*. Eur J Cancer.

[29] Scott, N.W., Fayers,P.,Aaronson,N.K.,Bottomley,A.,deGraeff,A.,Groenvold,M., EORTCQualityofLifeGroup, *EORTCQLQ-C30 Reference Values Manual. (2nded.) Brussels,Belgium.* 2008.

[30] Osoba D (1998). *Interpreting the significance of changes in health-related quality-of-life scores*. J Clin Oncol.

[31] Lagergren P (2007). *Health-related quality of life among patients cured by surgery for esophageal cancer*. Cancer.

[32] Blazeby JM (2000). *A prospective longitudinal study examining the quality of life of patients with esophageal carcinoma*. Cancer.

[33] van den Boorn, H.G., et al., *SOURCE: A Registry-Based Prediction Model for Overall Survival in Patients with Metastatic Oesophageal or Gastric Cancer.* Cancers (Basel), 2019. **11**(2). 10.3390/cancers11020187 PMC6406639 30764578

[34] Kauppila JH (2017). *Meta-analysis of health-related quality of life after minimally invasive versus open oesophagectomy for oesophageal cancer*. Br J Surg.

[35] Safieddine N (2009). *Health-related quality of life in esophageal cancer: effect of neoadjuvant chemoradiotherapy followed by surgical intervention*. J Thorac Cardiovasc Surg.

[36] Jezerskyte, E., et al., *Long-term health-related quality of life after McKeown and Ivor Lewis esophagectomy for esophageal carcinoma.* Dis Esophagus, 2020. 10.1093/dote/doaa022 PMC7672202 32444879

[37] Coebergh van den Braak RRJ (2018). *Nationwide comprehensive gastro-intestinal cancer cohorts: the 3P initiative*. Acta Oncol.

