# Supplementary material for: Long-Term Quality of Life Following Transthoracic and Transhiatal Esophagectomy for Esophageal Cancer
Source: J Gastrointest Surg. 2020 Sep 9;25(7):1657–66. doi: 10.1007/s11605-020-04783-4 (PMC8275507; doi:10.1007/s11605-020-04783-4)
Supplement: Supplementary file 1 — (DOCX 66 kb) [file 11605_2020_4783_MOESM1_ESM.docx]

### Supplementary table 1: Multivariable linear regression analysis of patients after transthoracic or transhiatal esophagectomy.

|  | **Covariates** | **B** | **95%CI** | | **p-value** |
| --- | --- | --- | --- | --- | --- |
|  |  |  | **Lower** | **Upper** |  |
| **Emotional functioning** | TTE | -3.0 | -13.683 | 7.761 | 0.586 |
|  | Age | 0.3 | -0.175 | 0.748 | 0.222 |
|  | Gender | 4.2 | -6.384 | 14.835 | 0.432 |
|  | Tumor location |  |  |  |  |
|  | Neoadjuvant treatment (yes/no) |  |  |  |  |
|  | Neoadjuvant therapy (CH/CRT) | -3.7 | -21.474 | 14.026 | 0.679 |
|  | Surgical approach | 5.5 | -5.818 | 16.800 | 0.339 |
|  | pT stage |  |  |  |  |
|  | Lymph node yield |  |  |  |  |
|  | Follow-up | 0.3 | 0.089 | 0.602 | **0.009** |
| **Social functioning** | TTE | 15.0 | 2.724 | 27.230 | **0.017** |
|  | Age | 0.7 | 0.118 | 1.191 | **0.017** |
|  | Gender | 8.0 | -4.276 | 20.219 | 0.200 |
|  | Tumor location |  |  |  |  |
|  | Neoadjuvant treatment (yes/no) |  |  |  |  |
|  | Neoadjuvant therapy (CH/CRT) | -9.7 | -30.339 | 10.939 | 0.354 |
|  | Surgical approach | 6.4 | -4.841 | 17.713 | 0.261 |
|  | pT stage |  |  |  |  |
|  | Lymph node yield |  |  |  |  |
|  | Follow-up |  |  |  |  |
| **Constipation** | TTE | -3.1 | -11.794 | 5.502 | 0.473 |
|  | Age | 0.2 | -0.123 | 0.591 | 0.196 |
|  | Gender | -7.2 | -15.666 | 1.215 | 0.093 |
|  | Tumor location |  |  |  |  |
|  | Neoadjuvant treatment (yes/no) |  |  |  |  |
|  | Neoadjuvant therapy (CH/CRT) | -7.6 | -21.475 | 6.353 | 0.284 |
|  | Surgical approach | -4.8 | -12.672 | 3.029 | 0.227 |
|  | pT stage | 1.3 | -1.153 | 3.745 | 0.297 |
|  | Lymph node yield | 0.1 | -0.226 | 0.412 | 0.564 |
|  | Follow-up |  |  |  |  |
| **Hair loss** | TTE | -29.4 | -49.108 | -9.671 | **0.004** |
|  | Age | -1.5 | -2.647 | -0.281 | **0.016** |
|  | Gender | -27.3 | -48.143 | -6.403 | **0.012** |
|  | Tumor location |  |  |  |  |
|  | Neoadjuvant treatment (yes/no) |  |  |  |  |
|  | Neoadjuvant therapy (CH/CRT) | 22.0 | -14.115 | 58.202 | 0.226 |
|  | Surgical approach |  |  |  |  |
|  | pT stage |  |  |  |  |
|  | Lymph node yield |  |  |  |  |
|  | Follow-up |  |  |  |  |

_Bold values represent significance. B=regression coefficient, CI=confidence interval. TTE is the reference to which THE is compared. CH = chemotherapy, CRT = chemoradiotherapy._

### Supplementary table 2: Subgroup univariable and multivariable linear regression analysis of HR-QoL after transthoracic and transhiatal esophagectomy in patients who received neoadjuvant treatment and who were operated minimally invasively.

|  |  |  |  | **Univariable analysis** | | | | **Multivariable analysis†** | | | |  |
| --- | --- | --- | --- | --- | --- | --- | --- | --- | --- | --- | --- | --- |
|  |  | **Transthoracic esophagectomy**  Mean (SD) | **Transhiatal esophagectomy** Mean (SD) | **B** | **95%CI** | | **p-value** | **B** | **95%CI** | | **p-value** | **Corrected p-value‡** |
|  |  | N(neo)=116 | N(neo)=34 |  |  |  |  |  |  |  |  |  |
|  |  | N(MI)=112 | N(MI)=22 |  | Lower | Upper |  |  | Lower | Upper |  |  |
| **EORTC QLQ-C30** | |  |  |  |  |  |  |  |  |  |  |  |
| Global Health | NT | 73.1 (19.7) | 75.3 (24.2) | 2.2 | -5.933 | 10.418 | 0.589 |  |  |  |  |  |
|  | MI | 72.9 (19.8) | 67.5 (24.7) | -5.4 | -15.113 | 4.280 | 0.271 |  |  |  |  |  |
| **Functioning** |  |  |  |  |  |  |  |  |  |  |  |  |
| Physical functioning | NT | 82.9 (19.6) | 76.6 (21.9) | -6.3 | -14.115 | 1.419 | 0.108 |  |  |  |  |  |
|  | MI | 82.5 (18.7) | 74.7 (21.1) | -7.8 | -16.679 | 0.984 | 0.081* | 13.8 | 2.755 | 24.933 | **0.015** | **0.030** |
| Role functioning | NT | 75.7 (26.5) | 72.2 (31.6) | -3.4 | -14.229 | 7.380 | 0.532 |  |  |  |  |  |
|  | MI | 74.5 (27.2) | 73.9 (31.7) | -0.6 | -13.536 | 12.305 | 0.925 |  |  |  |  |  |
| Emotional functioning | NT | 78.4 (23.9) | 84.1 (23.3) | 5.7 | -3.589 | 14.934 | 0.228 |  |  |  |  |  |
|  | MI | 78.3 (23.7) | 80.8 (23.4) | 2.5 | -8.634 | 13.594 | 0.660 |  |  |  |  |  |
| Cognitive functioning | NT | 83.4 (23.4) | 83.3 (14.4) | -0.1 | -8.567 | 8.424 | 0.987 |  |  |  |  |  |
|  | MI | 83.6 (23.3) | 84.1 (14.4) | 0.6 | -9.845 | 10.986 | 0.914 |  |  |  |  |  |
| Social functioning | NT | 79.7 (25.4) | 66.5 (35.2) | -13.1 | -26.203 | -0.081 | **0.049*** | 13.4 | 1.623 | 25.187 | **0.026** | 0.104 |
|  | MI | 79.0 (25.8) | 73.5 (32.8) | -5.5 | -17.963 | 6.947 | 0.383 |  |  |  |  |  |
| **Symptom scores** | |  |  |  |  |  |  |  |  |  |  |  |
| Fatigue | NT | 31.0 (27.1) | 32.7 (31.6) | 1.7 | -9.148 | 12.583 | 0.755 |  |  |  |  |  |
|  | MI | 32.0 (26.2) | 38.1 (29.8) | 6.1 | -6.272 | 18.497 | 0.331 |  |  |  |  |  |
| Nausea and vomiting | NT | 14.6 (23.0) | 10.1 (14.2) | -4.5 | -10.955 | 1.923 | 0.167 |  |  |  |  |  |
|  | MI | 14.9 (23.0) | 13.3 (19.7) | -1.6 | -11.990 | 8.776 | 0.760 |  |  |  |  |  |
| Pain | NT | 18.0 (24.8) | 14.7 (25.5) | -3.3 | -12.903 | 6.373 | 0.504 |  |  |  |  |  |
|  | MI | 17.3 (23.5) | 9.9 (17.6) | -7.4 | -17.870 | 3.032 | 0.163 |  |  |  |  |  |
| Dyspnea | NT | 28.2 (29.0) | 28.4 (31.9) | 0.3 | -11.174 | 11.715 | 0.963 |  |  |  |  |  |
|  | MI | 29.8 (29.1) | 22.7 (34.7) | -7.0 | -20.910 | 6.841 | 0.318 |  |  |  |  |  |
| Insomnia | NT | 26.8 (32.0) | 16.7 (26.3) | -10.1 | -22.010 | 1.752 | 0.094* | 6.3 | -7.221 | 19.748 | 0.360 | 1.440 |
|  | MI | 26.3 (31.1) | 15.2 (22.4) | -11.1 | -24.891 | 2.665 | 0.113 |  |  |  |  |  |
| Appetite loss | NT | 19.6 (30.0) | 15.7 (28.7) | -4.0 | -15.417 | 7.515 | 0.497 |  |  |  |  |  |
|  | MI | 19.3 (30.4) | 13.6 (26.5) | -5.7 | -19.415 | 8.099 | 0.417 |  |  |  |  |  |
| Constipation | NT | 7.9 (16.2) | 16.2 (23.7) | 8.3 | -0.625 | 17.159 | 0.068* | -4.6 | -12.819 | 3.662 | 0.274 | 1.096 |
|  | MI | 7.2 (15.2) | 15.9 (27.1) | 8.7 | -3.952 | 21.283 | 0.169 |  |  |  |  |  |
| Diarrhea | NT | 13.5 (20.1) | 16.2 (22.2) | 2.7 | -5.380 | 10.692 | 0.515 |  |  |  |  |  |
|  | MI | 16.1 (21.5) | 15.9 (25.0) | -0.2 | -10.560 | 10.163 | 0.973 |  |  |  |  |  |
| Financial difficulties | NT | 27.9 (29.3) | 34.3 (42.1) | 6.5 | -9.298 | 22.284 | 0.411 |  |  |  |  |  |
|  | MI | 28.3 (28.2) | 28.6 (39.8) | 0.3 | -18.469 | 19.097 | 0.973 |  |  |  |  |  |
| **EORTC QLQ-OG25** | |  |  |  |  |  |  |  |  |  |  |  |
| **Functioning** |  |  |  |  |  |  |  |  |  |  |  |  |
| Body image | NT | 77.4 (32.9) | 67.7 (39.4) | -9.7 | -23.059 | 3.633 | 0.152 |  |  |  |  |  |
|  | MI | 76.5 (33.9) | 83.3 (26.7) | 6.9 | -6.449 | 20.184 | 0.303 |  |  |  |  |  |
| **Symptom scores** | |  |  |  |  |  |  |  |  |  |  |  |
| Dysphagia | NT | 11.2 (16.9) | 16.3 (18.8) | 5.2 | -1.508 | 11.868 | 0.128 |  |  |  |  |  |
|  | MI | 11.1 (16.7) | 14.1 (19.8) | 3.1 | -4.879 | 11.041 | 0.445 |  |  |  |  |  |
| Eating | NT | 26.0 (24.7) | 29.2 (31.6) | 3.2 | -8.668 | 15.076 | 0.590 |  |  |  |  |  |
|  | MI | 26.3 (25.1) | 30.6 (35.1) | 4.3 | -11.873 | 20.461 | 0.589 |  |  |  |  |  |
| Reflux | NT | 22.6 (28.3) | 20.3 (23.0) | -2.2 | -12.715 | 8.268 | 0.676 |  |  |  |  |  |
|  | MI | 22.5 (28.7) | 23.1 (24.7) | 0.6 | -12.402 | 13.598 | 0.928 |  |  |  |  |  |
| Odynophagia | NT | 10.2 (20.9) | 9.8 (18.4) | -0.4 | -8.324 | 7.477 | 0.916 |  |  |  |  |  |
|  | MI | 10.8 (21.1) | 9.9 (19.0) | -1.0 | -10.621 | 8.683 | 0.843 |  |  |  |  |  |
| Pain and discomfort | NT | 17.1 (27.7) | 14.7 (19.6) | -2.4 | -12.477 | 7.671 | 0.638 |  |  |  |  |  |
|  | MI | 16.0 (27.2) | 13.6 (16.8) | -2.4 | -14.335 | 9.520 | 0.690 |  |  |  |  |  |
| Anxiety | NT | 31.4 (29.7) | 26.0 (32.4) | -5.4 | -17.133 | 6.286 | 0.362 |  |  |  |  |  |
|  | MI | 30.9 (28.6) | 25.8 (29.9) | -5.1 | -18.412 | 8.214 | 0.450 |  |  |  |  |  |
| Eating with others | NT | 14.5 (25.1) | 19.6 (28.6) | 5.2 | -4.885 | 15.192 | 0.312 |  |  |  |  |  |
|  | MI | 13.4 (24.2) | 18.2 (30.4) | 4.8 | -6.935 | 16.507 | 0.421 |  |  |  |  |  |
| Dry mouth | NT | 19.1 (27.1) | 13.7 (20.3) | -5.3 | -15.272 | 4.628 | 0.292 |  |  |  |  |  |
|  | MI | 18.2 (25.7) | 24.2 (29.4) | 6.0 | -6.205 | 18.212 | 0.332 |  |  |  |  |  |
| Trouble with taste | NT | 12.5 (22.4) | 8.8 (22.2) | -3.7 | -12.335 | 4.982 | 0.403 |  |  |  |  |  |
|  | MI | 13.2 (23.8) | 7.6 (22.8) | -5.6 | -16.579 | -16.579 | 0.311 |  |  |  |  |  |
| Trouble swallowing saliva | NT | 9.8 (21.0) | 9.9 (21.4) | -0.1 | -8.325 | 8.168 | 0.985 |  |  |  |  |  |
|  | MI | 10.6 (22.5) | 16.7 (26.7) | 6.1 | -4.692 | 16.813 | 0.267 |  |  |  |  |  |
| Choked when swallowing | NT | 15.8 (24.9) | 19.6 (29.7) | 3.8 | -6.260 | 13.928 | 0.096* | -9.0 | -20.272 | 2.227 | 0.115 | 0.460 |
|  | MI | 17.3 (25.2) | 19.7 (30.3) | 2.4 | -9.652 | 14.478 | 0.693 |  |  |  |  |  |
| Trouble with coughing | NT | 32.1 (31.9) | 23.5 (32.3) | -8.6 | -21.008 | 3.781 | 0.172 |  |  |  |  |  |
|  | MI | 34.9 (31.4) | 27.3 (36.6) | -7.6 | -22.544 | 7.337 | 0.316 |  |  |  |  |  |
| Trouble talking | NT | 8.1 (17.1) | 8.8 (17.0) | 0.7 | -5.896 | 7.323 | 0.831 |  |  |  |  |  |
|  | MI | 7.8 (17.0) | 1.5 (7.1) | -6.3 | -10.721 | -1.836 | **0.006*** | 7.3 | -2.504 | 17.171 | 0.142 | 0.284 |
| Weight loss | NT | 24.5 (30.6) | 20.0 (33.4) | -4.5 | -17.158 | 8.199 | 0.486 |  |  |  |  |  |
|  | MI | 23.2 (30.6) | 18.3 (33.3) | -4.9 | -19.850 | 10.066 | 0.519 |  |  |  |  |  |
| Hair loss | NT | 15.7 (30.0) | 29.2 (32.2) | 13.5 | -5.327 | 32.349 | 0.156 |  |  |  |  |  |
|  | MI | 15.7 (27.6) | 26.7 (36.5) | 11.0 | -16.964 | 38.987 | 0.430 |  |  |  |  |  |

_NT = neoadjuvant therapy. MI = minimally invasive esophagectomy. N (neo) = number of patients after neoadjuvant therapy. N(MI) = number of patients after minimally invasive surgery. Regression coefficient (B) with 95% confidence interval (CI) are shown for univariable and multivariable analysis. †=Corrected for confounders (Supplementary table 3 and 4). *=Health-related quality of life (HR-QoL) domains with p<0.1 in univariable analysis were entered in multivariable analysis. ‡= p-value corrected for multiple testing according to Bonferroni method._

### Supplementary table 3: Multivariable linear regression analysis of patients after minimally invasive transthoracic and transhiatal esophagectomy.

|  | **Covariates** | **B** | **95%CI** | | **p-value** |
| --- | --- | --- | --- | --- | --- |
|  |  |  | **Lower** | **Upper** |  |
| **Physical functioning** | TTE | 13.8 | 2.755 | 24.933 | **0.015** |
|  | Age | 0.1 | -0.307 | 0.552 | 0.572 |
|  | Gender | 3.0 | -7.756 | 13.844 | 0.578 |
|  | Neoadjuvant treatment (yes/no) |  |  |  |  |
|  | Neoadjuvant therapy (CH/CRT) | -8.4 | -28.280 | 11.473 | 0.404 |
|  | cN stage |  |  |  |  |
|  | Lymph node yield |  |  |  |  |
| **Trouble talking** | TTE | 7.3 | -2.504 | 17.171 | 0.142 |
|  | Age | 0.1 | -0.248 | 0.533 | 0.471 |
|  | Gender | -2.6 | -12.162 | 7.015 | 0.596 |
|  | Neoadjuvant treatment (yes/no) |  |  |  |  |
|  | Neoadjuvant therapy (CH/CRT) | -4.8 | -22.426 | 12.865 | 0.592 |
|  | cN stage |  |  |  |  |
|  | Lymph node yield |  |  |  |  |

_Bold values represent significance. B = regression coefficient. CI = confidence interval. CH = chemotherapy. CRT = chemoradiotherapy._

### Supplementary table 4: Multivariable linear regression analysis of patients after transthoracic and transhiatal esophagectomy who received neoadjuvant treatment.

|  | **Covariates** | **B** | **95%CI** | | **p-value** |
| --- | --- | --- | --- | --- | --- |
|  |  |  | **Lower** | **Upper** |  |
| **Social functioning** | TTE | 13.4 | 1.623 | 25.187 | 0.026 |
|  | Age | 0.6 | 0.103 | 1.174 | 0.020 |
|  | Gender | 8.3 | -3.951 | 20.497 | 0.183 |
|  | Follow-up |  |  |  |  |
|  | ASA classification |  |  |  |  |
|  | Surgical approach | 6.3 | -5.009 | 17.520 | 0.274 |
|  | pT stage |  |  |  |  |
|  | Lymph node yield |  |  |  |  |
| **Insomnia** | TTE | 6.3 | -7.221 | 19.748 | 0.360 |
|  | Age | -0.5 | -1.137 | 0.064 | 0.080 |
|  | Gender | -4.0 | -17.913 | 9.937 | 0.572 |
|  | Follow-up |  |  |  |  |
|  | ASA classification |  |  |  |  |
|  | Surgical approach | -5.0 | -18.080 | 8.018 | 0.447 |
|  | pT stage |  |  |  |  |
|  | Lymph node yield | 0.4 | -0.126 | 0.921 | 0.135 |
| **Constipation** | TTE | -4.6 | -12.819 | 3.662 | 0.274 |
|  | Age | 0.2 | -0.134 | 0.579 | 0.219 |
|  | Gender | -7.2 | -15.634 | 1.255 | 0.095 |
|  | Follow-up |  |  |  |  |
|  | ASA classification |  |  |  |  |
|  | Surgical approach | -5.1 | -12.957 | 2.712 | 0.198 |
|  | pT stage | 1.4 | -1.061 | 3.829 | 0.265 |
|  | Lymph node yield | 0.1 | -0.183 | 0.441 | 0.415 |
| **Choked when swallowing** | TTE | -9.0 | -20.272 | 2.227 | 0.115 |
|  | Age | -0.2 | -0.696 | 0.334 | 0.488 |
|  | Gender | -12.3 | -23.816 | -0.699 | 0.038 |
|  | Follow-up |  |  |  |  |
|  | ASA classification |  |  |  |  |
|  | Surgical approach | 9.0 | -1.798 | 19.852 | 0.101 |
|  | pT stage | -2.0 | -5.557 | 1.464 | 0.251 |
|  | Lymph node yield |  |  |  |  |

_Bold values represent significance. B = regression coefficient, CI = confidence interval. CH = chemotherapy. CRT = chemoradiotherapy._
